# Management of Extensive Maxillofacial Trauma With Bony Foreign Body Within the Orbit From a Chainsaw Injury

**Published:** 2011-11-21

**Authors:** Randall O. Craft, Kyle R. Eberlin, Michael H. Stella, Edward J. Caterson

**Affiliations:** From the ^a^Division of Plastic Surgery, Department of Surgery; ^b^Department of Radiology, Brigham and Women's Hospital, Harvard Medical School, Boston, MA

## Abstract

**Objective:** The goal of this case report is to characterize injury patterns typical for chainsaw injuries to the face. We describe our approach to the soft tissue and skeletal injury patterns seen in these injuries. **Methods:** We present a case report of a traumatic chainsaw injury to the face. **Results:** A literature review of the typical injury patterns seen in chainsaw injuries to the face is discussed. Fractures to the bony orbit are on of the most common findings. Traumatic orbital fractures are often associated with other facial fractures, including those of the maxillary sinus and naso-orbital-ethmoid (NOE) region. There is a reported 47% incidence of lacrimal obstruction after NOE fractures, most caused by bone malposition or damage to the lacrimal sac or duct. Misdiagnosis of this injury pattern can lead to chronic patient morbidity. **Conclusion:** We present a case of traumatic orbital fracture with subsequent bony intrusion into the orbit, necessitating urgent exploration. The compound soft tissue and skeletal injury in this patient is typical for patients with associated lacrimal injury. Awareness of the injury patterns and treatment algorithms of these cases allows for appropriate assessment and intervention.

Most chainsaw injuries are accidental, resulting from an inexperienced operator or an improperly guarded blade. Although epidemiological data have suggested a lower prevalence of maxillofacial injury compared to other body parts, the incidence of these injuries has increased over recent years.^1^^,^^2^ The resultant injuries may be complex and involve both the soft tissue and facial skeleton.^3^ The chainsaw teeth produce a characteristic laceration given the serrated nature of the blade, causing avulsion with tissue loss coincident with chain width of 8 to 10 mm at a minimum. Projecting anatomical parts are often snared in the upward moving chain, and include the eyebrow, malar eminence, nose, lips, and free edge of the upper eyelid. Conversely, injury to the globe and lower lid is rare.^4^

Fractures of the orbit are far more common and often involve the orbital floor and/or rim. These fractures can occur internally in isolation or in conjunction with fractures of the orbital rim. Associated subjective diplopia can result from muscle contusion, incarceration, or displacement of the globe 1 cm beyond its normal anatomical position, resulting in diplopia. Traumatic orbital fractures are often associated with other facial fractures, including those of the maxillary sinus and naso-orbital-ethmoid (NOE) region. There is a reported 47% incidence of lacrimal obstruction after NOE fractures, most caused by bone malposition or damage to the lacrimal sac or duct.^5^ Extensive damage to the lacrimal system can lead to epiphora, with the potential for recurrent infection and visual impairment. In cases where there is inadequate tissue for reconstruction (<8 mm of patency from the punctum), lacrimal canalicular bypass surgery may be warranted.^6^^-^8 In this case, we review a patient with a complex facial injury resulting from a chainsaw kickback to the left face. The resulting soft tissue injury and fracture pattern resulted in displacement of a portion of the medial buttress into the orbit, causing globe proptosis and ocular muscle entrapment with simultaneous destruction of the lacrimal system.

## CASE REPORT

**Figure 1:**
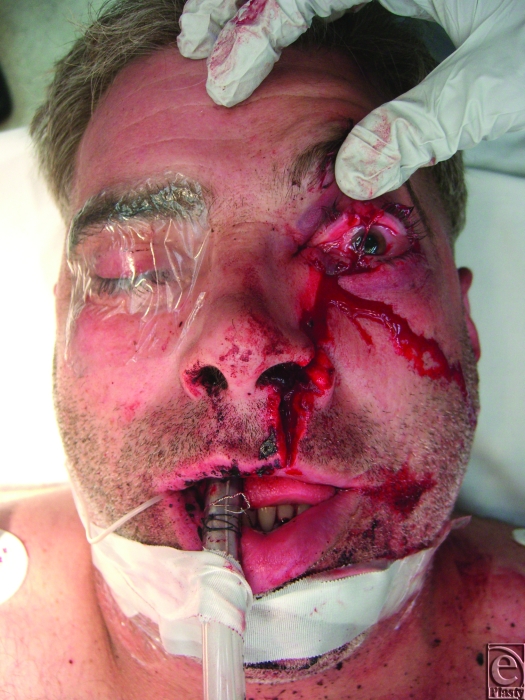
Preoperative clinical appearance.

The patient is a 41-year-old man who presented to the emergency department with a 12-cm vertically oriented left facial laceration resulting from a chainsaw (Fig 1). The laceration extended from the height of the left cupid bow on the upper lip, through the left alar base, to the left superior eyelid as seen in Figure 1. Although the patient was not able to withstand a thorough examination in the emergency department due to pain, anxiety, and acute alcohol intoxication, his visual acuity was found to be grossly intact in the left eye. Tonometry revealed an elevated intraocular pressure of 26 mm Hg on the left compared to 18 mm Hg in the right uninjured orbit. He had clinical signs of proptosis with significant horizontal gaze restriction indicating ocular muscle entrapment most prominent in lateral and medial gaze.

**Figure 2:**
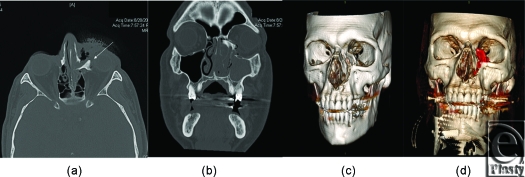
Computed tomography scan demonstrating injury pattern (a) axial view with unilateral nasal orbital ethmoid (NOE) segment rotated 180° into ectopic position, (b) coronal view demonstrating the ectopic bone from left nasal piriform rotated posteriorly, (c) 3D reconstruction of the bony injury left maxilla, and (d) red indicates the 22-mm segment of bone rotated posteriorly into the orbit. This ectopic bone was interposed between the inferior oblique and the medial rectus muscles of the left globe.

A maxillofacial computed tomographic scan with axial, coronal, and sagittal images was obtained to determine the presence and extent of bony injury. This demonstrated a unilateral left naso-oribital ethmoidal fracture, with displacement of the medial buttress of the left nasal aperture into the inferiomedial left orbit. This 22-mm segment was rotated approximately 180° about the craniocaudal axis (Figs 2a and 2b). Radiographically, the fragment elevated and tented the inferior oblique muscle. A large osseous gap was seen in the medial floor of the left orbit at the expected location of the distal lacrimal duct and sac. There was also a complex fracture of the anteromedial wall the left maxillary sinus with associated hemosinus and disruption of the left nasal turbinates.

Given his proptosis, ocular entrapment, and extent of soft tissue injury, the patient was taken for urgent operative exploration. A forced duction test was performed intraoperatively which revealed movement only in a medial direction with otherwise completely restricted movement. Further exploration of the laceration revealed a large intervening bone fragment, which originated from the piriform/medial buttress of the left maxilla and was rotated 180° into the orbit. The fragment was reduced and secured in place with a 1.3-mm plate. The naso-orbital ethmoid complex was explored through a modified Lynch incision, and the medial canthal tendon mechanism was found to be stable. However, the entire left lacrimal drainage system appeared to be irrevocably destroyed. The left inferior oblique muscle was found to be lacerated and avulsed from its bony origin; this was repaired and replaced to proper anatomic position. A 1.3-mm plate was used to reconstruct the bone defect at the infra-orbital rim. After fixation, forced duction test revealed improved ocular mobility and resolution of proptosis. The laceration was closed in layers.

**Figure 3:**
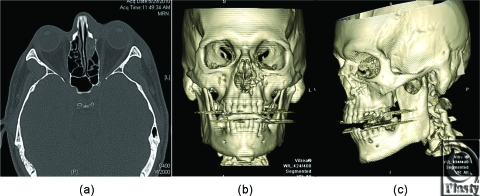
Postoperative maxillofacial computed tomographic scan with 3D reconstruction (a) axial view, (b) anteroposterior 3D view, (c) oblique 3D view.

**Figure 4:**
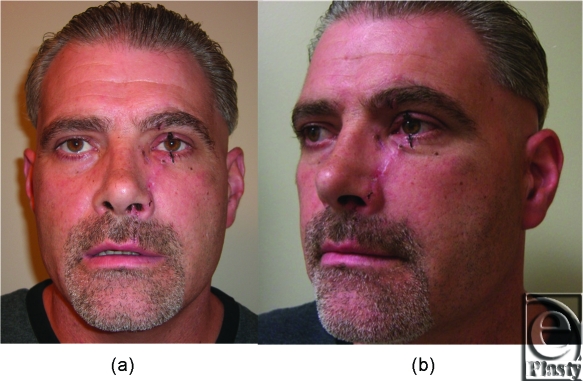
(a) Two weeks following injury the patient has shown good healing without orbital dystopia or early enopthalmus. (b) Lateral view demonstrates appropriate orbital position (tarsal plate sutures are still present at the eyelid margins).

Postoperative computed tomographic scan obtained on the following day showed reduction of the bone fragment with no residual foreign body in the globe or evidence muscle impingement (Figs 3a–3c). Subsequent follow-up in clinic 2 weeks postoperatively demonstrated an acceptable aesthetic result without orbital dystopia or enophthalmos (Figs 4a and 4b). In addition, the patient had full return of extraocular motility as determined by provocative testing.

## DISCUSSION

Maxillofacial trauma resulting from chainsaw injury, although described only rarely in the literature, is becoming more common with the increased use of this equipment by untrained individuals at home or professional arborists. A recent epidemiologic study of 133 patients who suffered saw injuries to the maxillofacial region over 19 years revealed that all patients were male, with the largest number injured between the ages of 31 and 40.^4^ All cases were accidental and self-inflicted. Isolated soft tissue injuries occurred in 70% of the patients, with 30% having involvement of the facial skeleton. Seventy percent of the injuries were localized to the left side, presumably the result of geometry associated with a right-handed user. The mechanism of injury with the chainsaw can be explained by reactive forces (kickback, pushback, and pull-in) during chainsaw action that occurs by its sudden break in contact with a hard medium, launching the saw rapidly backward toward the operator.^4^

Although 10% of patients in this series had orbital fractures associated with chainsaw injuries, none had bony foreign bodies or displacement of the globe from its normal anatomic position and underwent standard treatment according to maxillofacial trauma principles. Furthermore, while 30% of patients had compound soft tissue and facial skeleton injuries, there was no report of injury to the lacrimal system. (3) Our patient, notably, was found to have a bony foreign body in the orbit with significant displacement of the globe and this prompted emergent exploration and reduction. The pattern of NOE fracture and soft tissue injury also raised a high suspicion of associated lacrimal system damage.

The nasolacrimal duct originates a few millimeters posterior to the medial canthal tendon and courses from the lacrimal sac inferiorly and posteriorly before becoming enclosed in the bony canal and draining through the valve of Hasner into the inferior meatus of the nose. Loss of patency disrupts the normal tear drainage mechanism and can lead to multiple complications, including visual impairment.^9^ There is controversy regarding the optimal treatment of a disrupted lacrimal system in the setting of trauma, especially pertaining to the practice of nasolacrimal intubation.^10^^,^^11^ There have been reports of exploratory probing at the time of surgery damaging an intact system or exacerbating an existing injury, and as a result, some authors favor delayed assessment and repair.^11^ Conversely, delayed assessment of the lacrimal system without initial intervention can result in cicatricial obstruction, necessitating secondary repair which is more difficult.^9^ Certain authors have advocated prophylactic intubation in the setting of complex injuries involving the medial canthal tendon and the bony complex that encases the distal system to obviate the need for secondary lacrimal bypass surgery.^9^

In the case of irreparable injury due to extensive tissue loss, as in the present case, eventual conjunctivodacryocystorhinostomy with the insertion of a Jones tube may be required in order to avoid the long-term sequelae of lacrimal dysfunction, including recurrent dacryocystitis, visual impairment, and impaired quality of life. (A8) Complications, however, often occur following conjunctivodacryocystorhinostomy, requiring tube replacement, repositioning of the tube, and cleaning over an indefinite period of time.^6^ Despite this, patients report a high level of long-term satisfaction if they are able to obtain a comfortable, dry eye.^6^ At 2 months follow-up, our current patient was not yet symptomatic with epiphora to pursue additional evaluation or surgical correction.

Although historically uncommon, maxillofacial injuries from chainsaw have been reported with increasing frequency. Early intervention with operative debridement of devitalized soft tissue and reduction of bony fractures remain the mainstay of treatment. In this case, our patient had a displaced, entrapped globe from bony distortion of the orbit warranting urgent decompression and fracture reduction. With such injuries in the setting of NOE fractures, we believe that clinicians should have a high suspicion for damage to the lacrimal system although the initial management remains controversial.
